# Deep-learning-based gas identification by time-variant illumination of a single micro-LED-embedded gas sensor

**DOI:** 10.1038/s41377-023-01120-7

**Published:** 2023-04-18

**Authors:** Incheol Cho, Kichul Lee, Young Chul Sim, Jae-Seok Jeong, Minkyu Cho, Heechan Jung, Mingu Kang, Yong-Hoon Cho, Seung Chul Ha, Kuk-Jin Yoon, Inkyu Park

**Affiliations:** 1 Department of Mechanical Engineering, Korea Advanced Institute of Science and Technology (KAIST), 291 Daehak-ro, Yuseong-gu, Daejeon, 34141 Republic of Korea; 2 Department of Physics, Korea Advanced Institute of Science and Technology (KAIST), 291 Daehak-ro, Yuseong-gu, Daejeon, 34141 Republic of Korea; 3 SENKO Co., Ltd., 485, Oesammi-Dong, Osan-Si, Gyeonggil-Do 18111 Republic of Korea

**Keywords:** Lasers, LEDs and light sources, Electronics, photonics and device physics

## Abstract

Electronic nose (e-nose) technology for selectively identifying a target gas through chemoresistive sensors has gained much attention for various applications, such as smart factory and personal health monitoring. To overcome the cross-reactivity problem of chemoresistive sensors to various gas species, herein, we propose a novel sensing strategy based on a single micro-LED (μLED)-embedded photoactivated (μLP) gas sensor, utilizing the time-variant illumination for identifying the species and concentrations of various target gases. A fast-changing pseudorandom voltage input is applied to the μLED to generate forced transient sensor responses. A deep neural network is employed to analyze the obtained complex transient signals for gas detection and concentration estimation. The proposed sensor system achieves high classification (~96.99%) and quantification (mean absolute percentage error ~ 31.99%) accuracies for various toxic gases (methanol, ethanol, acetone, and nitrogen dioxide) with a single gas sensor consuming 0.53 mW. The proposed method may significantly improve the efficiency of e-nose technology in terms of cost, space, and power consumption.

## Abstract (web summary)

In this study, a novel sensing strategy to identify gas species selectively and to estimate the concentrations of multiple gases was proposed. Highly sensitive gold NPs coated GLAD In_2_O_3_ was used as a gas-sensing material, and time-variant pseudorandom illumination of a single monolithic micro-LED (μLED) photoactivated (μLP) gas sensor was applied. Transient sensor signals, owing to the rapid changes in the light intensity of μLED and different reaction kinetics of various gas species, facilitate the identification of gas species. A deep convolutional neural network (CNN) was used to effectively analyze the complex frequency spectrogram of the transient sensor signals. As such, the identification of four mono-gas environments and binary gas mixtures (mixed ethanol and methanol) were successfully demonstrated with high accuracy. The total power consumption of the μLP was only 0.53 mW, one-hundredth of the conventional electronic nose (e-nose) system. Therefore, the proposed method may significantly improve the efficiency of e-nose technology in terms of cost, space, and power consumption.

## Graphical abstract

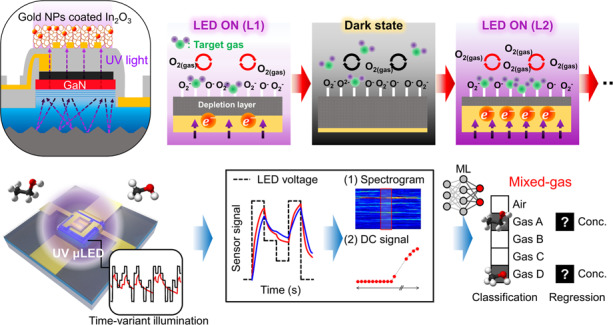

## Introduction

Advances in mobile gas monitoring technology have resulted in many emerging applications, such as smart factories, smart agriculture, personalized health-monitoring, and internet-of-things (IoT) appliances. Chemoresistive gas sensors are one of the most promising components for the future gas monitoring systems because of their high sensitivity, compact size, simple measurement, and low cost^1^. Accordingly, semiconductor metal oxides (SMOs)^2–4^, carbon nanomaterials (e.g., graphene and carbon nanotubes)^5–7^, conductive polymers^8,9^, and functionalized silicon^10–12^ have been thoroughly studied as potential candidates for chemoresistive sensing materials. Despite various attempts, chemoresistive gas sensors have suffered from the selectivity problem attributed to the nonspecific responses of those sensors to most reactive gas species, thereby resulting in challenges to accurately estimate the type and concentration of a specific gas. Accordingly, numerous studies on specific nanostructures/composites-based sensing materials and surface modification with catalysts have been reported to improve the selectivity of chemoresistive gas sensors^3,13–17^. These sensing materials exhibit a higher response to a specific target gas relative to the other interfering gases. Additionally, gas-selective filters have been introduced^18–20^. They are placed outside the sensor package or directly coated on the sensing materials to facilitate only the desired gas molecules to approach the sensor. However, because these approaches cannot fundamentally eliminate the cross-reactivity of gas sensors to various gas species, their practical uses in gas identification are limited.

Electronic nose (e-nose) technologies have emerged to overcome these selectivity problems. General e-nose systems are composed of multiple gas sensor arrays, each exhibiting a different reactivity to specific gases. Similar to diverse olfactory cells and acceptors in an animal nose, gas species can be identified by analyzing the patterns of the multi-sensor signals^21^. To date, various e-nose systems, such as arrays of commercial packaged gas sensors^22^ and highly integrated micro/nano-sensors^23–25^, have been introduced. In addition, applications of signal processing and machine-learning (ML) algorithms, such as principal component analysis (PCA)^26–29^, support vector machine (SVM)^24,25^, Gaussian mixture model (GMM)^30^, k-nearest neighbors (KNN)^23^, and neural-network-based ML algorithms^31–33^, have been extensively studied in this field. Nevertheless, e-noses using multi-sensors have critical drawbacks: the cost, power consumption, and volume of the entire system proportionally rise with the increase in the number of used gas sensors.

In this study, we propose a novel gas-identification strategy based on a single micro light-emitting diode (μLED)-embedded photoactivated (μLP) gas sensor, utilizing the time-variant illumination, coupled with a deep-learning-based analysis. In our previous study, we developed ultra-low power μLP gas sensors and verified their high gas-sensing performance under consistent illumination conditions^34^. Moreover, the excellent mechanical stability and microsecond-level latency of the gallium nitride (GaN)-based μLED facilitate rapid changes in the light intensity. Under the changes in light intensity, chemical activations and deactivations are repeated on the surface of the SMOs. The resulting temporal transient signals can accordingly reflect specific patterns depending on the different gas species owing to the differences in their reaction kinetics. We demonstrate this concept using various concentrations of multiple gas species (methanol, ethanol, acetone and nitrogen dioxide), with pseudorandom operation of the μLED. In addition, the deep convolutional neural network (D-CNN) decodes the complex frequency spectrogram of the transient sensor signals and predicts the gas species as well as estimates the concentrations. This dual-task performance of the D-CNN facilitates successful real-time identification of mono-gas environments and binary gas mixtures. Thus, the proposed strategy is expected to facilitate efficiency in terms of cost, space, and power consumption and is applicable to the analysis of various gas environments.

## Results

### Description and characterization of the μLP gas sensor

**Fig. 1:**
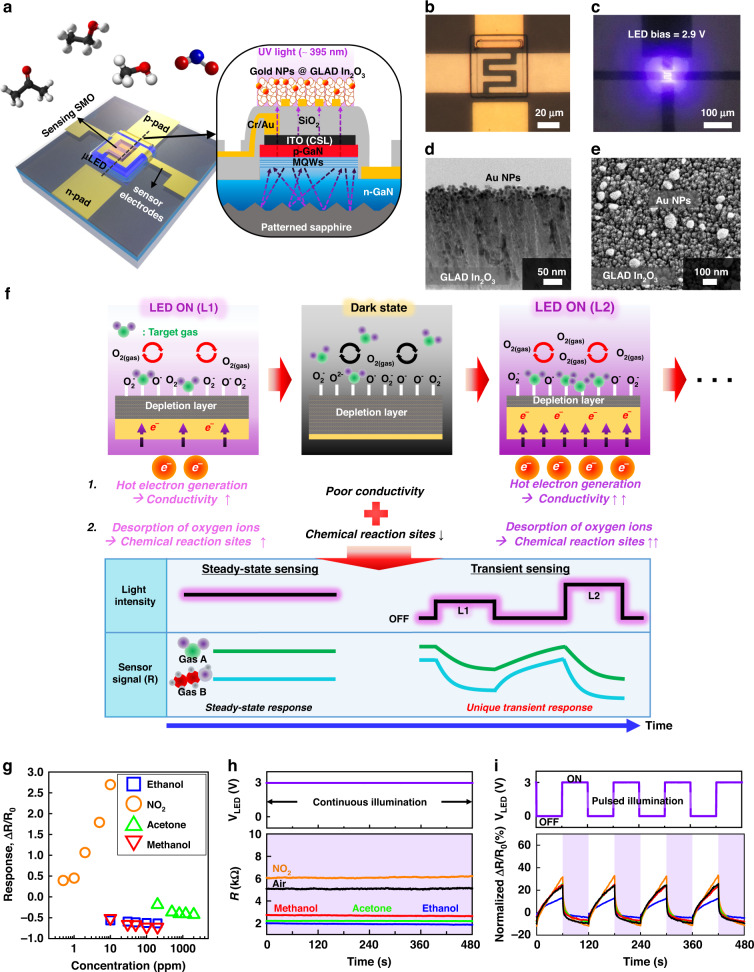
Description and characterization of sensor device. **a** Schematic illustration of a micro-LED (μLED)-embedded photoactivated (μLP) gas sensor device. The inset illustrates a cross-sectional structure of the sensor device. **b**, **c** Optical microscopic images of the fabricated sensor and near ultraviolet light-emitting μLED (λ_peak_ = 395 nm) with forward bias = 2.9 V. **d**, **e** Transmission electron microscopy and scanning electron microscopy images of cross-sectional and top views of gold NPs coated porous, columnar indium oxide (In_2_O_3_) sensing film deposited by a glancing angle deposition (GLAD) method. **f** Schematic illustration explaining the mechanism and advantages of pulsed illumination operation. L2 has a stronger light intensity than L1. By using random pulsed illumination instead of the steady illumination of μLEDs, more unique response data can be generated from each target gas within the same amount of time, enabling selective gas discrimination with a single sensor. **g** Calibration curves of the μLP gas sensor with representative reducing gases such as methanol (CH_3_OH), ethanol (C_2_H_5_OH), acetone (CH_3_COCH_3_), and nitrogen dioxide (NO_2_) under continuous illumination of μLED (forward bias = 3 V). **h** Dynamic sensor responses (*R* and *ΔR/R*_0_) to 10-ppm methanol, 10-ppm ethanol, 200-ppm acetone, and 0.5-ppm NO_2_ under continuously illuminating state and **i** on-off alternating state of μLED

The structure of the μLP gas sensor is illustrated in Fig. 1a. It has two *p*-*n* contact electrodes for applying forward bias to the μLED and two interdigitated electrodes on the surface of the device for measuring the conductance of the SMO-sensing materials. The inset image in Fig. 1a shows the cross-sectional structure of the sensor in detail. Epi-layers of *n*-GaN, multi-quantum wells (MQWs), and *p*-GaN are grown by a well-defined metal-organic chemical vapor deposition (MOCVD) process. The detailed fabrication process is described in the Materials and Methods and Fig. S1. The emission spectrum of the fabricated μLED is set in the near ultraviolet (UV) light range (*λ*_peak_ = 395 nm and full width at half maximum (FWHM) = 14 nm) where Al(In)GaN LEDs are known to have a high energy efficiency^35^. Furthermore, the extremely small gap (the thickness of the SiO_2_ insulation layer is approximately 1 µm) between the light source and the sensing material minimizes the energy loss. The light-emitting performance of the fabricated μLED device is provided in Fig. S2. The nano-porous indium oxide (In_2_O_3_) sensing film is deposited on the surface electrodes through the glancing angle deposition (GLAD) technique. GLAD is a technique in which the direction of vapor flux impinging on a substrate is inclined, and the substrate rotates simultaneously during the deposition^36^. By the nanoscale shadowing effect, porous, columnar, and granular metal oxide films can be formed. After the GLAD process, gold nanoparticles (NPs) were coated on the In_2_O_3_ surface by e-beam evaporation. When the light is illuminated on the plasmonic metal NPs, localized surface plasmon resonance (LSPR) occurs and hot electrons are generated. These electrons can be transferred from metal NPs to metal oxide, improving the gas sensitivity, response and recovery speed. Figure 1b–e show the results of the device fabrication. Figure 1b, c shows the optical microscopic images of the fabricated sensor device and the light-emitting state of the sensor under a forward bias of 2.9 V, respectively. The emission area is designed to be 50 × 50 μm^2^. Figure 1d, e demonstrates the transmission electron microscope (TEM) image of the cross-section-view and scanning electron microscopy (SEM) image of the top-view of the GLAD In_2_O_3_ sensing material coated with gold NPs, respectively. The nano-porous sensing films were well-formulated with a thickness of approximately 268 nm and a porosity of approximately 59.9% ((*top-view area of voids*)/(*total top-view area*) × 100 (%) from Fig. 1e, assuming that the film has a prismatic columnar structure), while the mean diameter of the nanocolumns is 30.4 nm (Fig. S3). The average diameter of the e-beam coated gold NPs was 10 nm. As shown in Fig. S4, higher porosity film can be obtained at higher tilting angles (*θ* = 85°) due to the greater nanoscale shadowing effect, and this exhibits the highest and fastest sensor responses over most concentration ranges. Therefore, the condition for maximizing the porosity was selected so that the sensor signal can make significant dynamic changes while changing the light intensity. The electrical resistance of In_2_O_3_, an n-type semiconductor, decreases when it comes in contact with reducing gases, such as methanol (CH_3_OH), ethanol (C_2_H_5_OH), and acetone (CH_3_COCH_3_), owing to the reduced density of the adsorbed oxygen ions and the thinned surface electron-depletion layer. In contrast, the electrical resistance of In_2_O_3_ increases when exposed to oxidizing gases, such as nitrogen dioxide (NO_2_). Here, the consistent photoactivation generates reactive hot electrons and promotes surface redox reactions of gas analytes. After a sufficient interval, upon introduction to a gas environment, the sensor reaches an equilibrium state, maintaining a steady sensor signal. On the other hand, Fig. 1f. illustrates a novel technique using random pulsed illumination of μLED. When the μLED is turned on, electrical conductivity is raised by hot electron generation, and desorption of adsorbed oxygen on the surface of metal oxide is promoted, increasing the reaction site with the target gas. The higher the light intensity, the more active this reaction is, and in the presence of each target gas, a different and unique transient sensor signal can be included in the sensor’s transient signal. Therefore, by using pulsed illumination instead of steady illumination, more diverse data can be obtained within the same time period, enabling selective gas detection with only a single sensor. The steady-state responses (Δ*R*/*R*_0_) of the fabricated sensor to the above-mentioned reducing gases are summarized in Fig. 1g. These response curves were acquired under consistent light illumination by the embedded μLED (*V*_LED_ = 3V). In Fig. 1g, the response span for the tested gases overlaps in specific concentration ranges of each gas. Figure 1h shows the cross-sensitivity of the sensor to methanol (10 ppm), ethanol (10 ppm), and acetone (200 ppm) under consistent illumination (*V*_LED_ = 3V) more clearly. In conclusion, it is difficult to identify the specific gas species and concentrations using only a single sensor signal in an unknown gas environment. To resolve this problem, our novel strategy focuses on the different reaction kinetics of different gases. Generally, the reaction speed of gases at the surface of SMOs can be increased by supplying external activation energy (heat or photon energy). Here, the reaction kinetics are also influenced by the intrinsic chemical/physical characteristics of specific gas molecules, such as the adsorption energy, dissociation energy, and surface diffusivity. By periodically changing the light intensity and modulating the μLED pulses, the sensor can always be in a non-steady state. While the steady-state responses were similar, as shown in Fig. 1h, the temporal transient responses differed depending on the different tested gases, as shown in Fig. 1i. Therefore, this approach provides an opportunity to identify gas species and estimate gas concentrations by monitoring the distinctive temporal patterns of a single sensor signal.

### Pseudorandom operation results of μLED

**Fig. 2:**
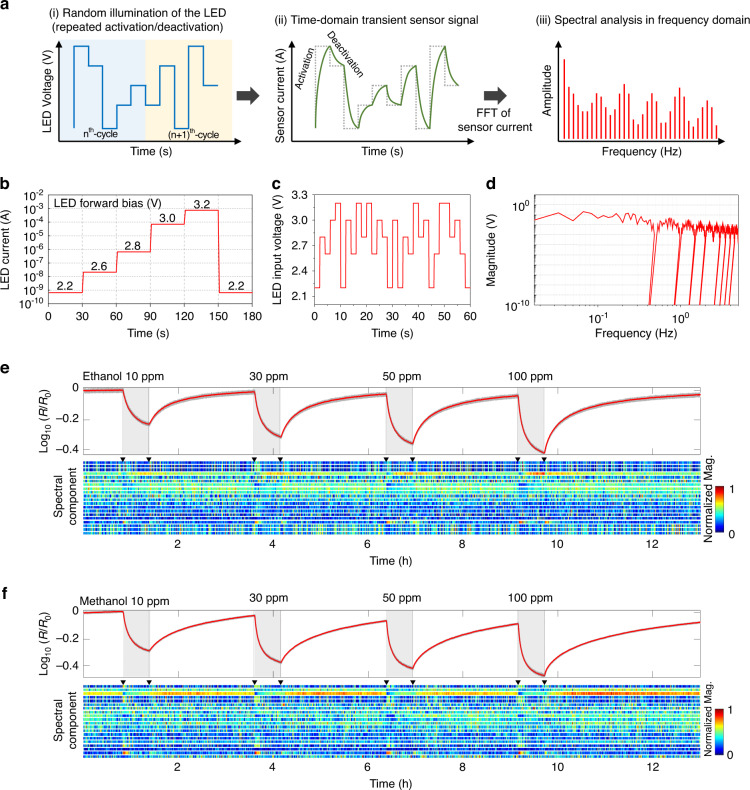
Investigation of pseudorandom operation. **a** Schematic of the operation strategy of the micro-LED (μLED)-embedded photoactivated gas sensor. **a**-(i) shows a pseudorandom input to μLED consisting of shuffled five levels of voltages. **a**-(ii) shows the time-domain transient sensor signal (sensor current). Alternation of photoactivation and deactivation causes the difference in the transient signals to different gas species. **a**-(iii) shows the frequency spectrum of the normalized sensor signal. **b** Measured forward current of μLED with five selected levels of μLED voltages. **c** Pseudorandom input consisting of five selected levels of voltages (time interval = 2 s and sampling rate = 10 Hz) in a unit time window (60 s). **d** Frequency spectrum of pseudorandom input in a time window. **e**, **f** Dynamic response (Log_10_(*R*/*R*_0_)) and spectrogram of the sensor signal to ethanol and methanol with various concentrations. Gray and red lines indicate raw transient signals and moving averages in a 60 s time window, respectively. The highly ranked 46 spectral components were used for the spectrogram data, but only 20 components are shown in the figure

Figure 2a shows the schematic process-flow of the sensor operation. As shown in Fig. 2a-(i), a pseudorandom input is applied to the embedded μLED. The pseudorandom input is composed of randomly shuffled voltages with five different levels and uses a fixed time interval for each level. A similar operational concept was first attempted by a previous study that utilized microheater-based SMO gas sensors^37,38^. The pseudorandom input is more advantageous to the generation of transient sensor signals than monotonous wave inputs such as sine, triangular, and square waves (Refer to Fig. S5 illustrating various input waveforms and their frequency spectra). The pseudorandom signals contain all frequency components, similar to a white noise; investigating the most advantageous operating frequency for gas discrimination in advance is not generally required. However, when applying such pseudorandom operation to microheater-based gas sensors, the modulation of temperature should be restricted to a small range, and the transition of temperature should be adequately slow to avoid a thermal shock and mechanical fracture of microheaters. In contrast, the excellent durability of GaN-based μLED facilitates more dramatic, wide-range alternation of light intensity and reliable long-term use in practical fields. Figure 2a-(ii) shows the time-domain transient sensor response (particularly, sensor current) under the pseudorandom operation of μLED. During the increase in the light intensity, the sensor current also increases owing to the generation of photo-carriers, while the surface of SMO is chemically activated. In contrast, the sensor current drops and the activated surface partially degenerates when the light intensity decreases. For both activation and deactivation steps, both the quick physical response (photo-current) and the relatively slow chemical response (gas reaction at the surface) of SMO contribute to the transient sensor signal. In Fig. 2a-(iii), the transient sensor signal is analyzed by transforming the sensor signal to the frequency domain. Prior to calculating the frequency spectrum, the sensor signal (*I*_sensor_) is normalized ((*X* - *μ*)/*σ*, where *X* is the raw signal) by the mean (*μ*) and standard deviation (*σ*) in the fixed time window (60 s). The distinctive patterns originating from differences in the chemical kinetics of various gas reactions can be obtained from the frequency spectrum.

Figure 2b shows the measured forward current of the fabricated μLED with 5 selected voltage levels (2.2, 2.6, 2.8, 3.0, and 3.2 V). The turn-on voltage of the fabricated μLED is 2.4 V; the μLED is completely turned off at *V*_LED_ = 2.2 V. Thus, five voltage levels of the pseudorandom input are composed of four on-state voltage levels and one off-state voltage levels. In addition, the time interval for switching between the different voltage levels is set to 2 s (switching frequency = 0.5 Hz and sampling frequency = 10 Hz). The pseudorandom input is generated by shuffling five selected voltage levels in a unit period (2 s × 5 = 10 s). This process is precisely controlled by an interface software connected to a voltage source. Figure 2c shows the generated pseudorandom input in a time window of 60 s. Each voltage level appears with the same probability in the time window. The average power consumption of the μLED, calculated using the pseudorandom input voltages and their measured μLED current in Fig. 2b, is only 526 μW. Figure 2d shows the frequency spectrum of the pseudorandom input in Fig. 2c. The upper bound frequency is 5 Hz, which is half of the sampling frequency (*f*_s_ = 10 Hz), according to Nyquist theorem; the spectral resolution is *f*_s_/*L* = 1/60 Hz, where *L* is the length of the time frame of the pseudorandom input. As mentioned before, the frequency spectrum of the pseudorandom input contains almost all frequency components, similar to the power spectrum of a white noise. Exceptionally, there are a few cut-off regions at multiples of 0.5 Hz that originate from the switching interval (2 s) of the multi-voltage levels.

Sensing data corresponding to the various concentrations of methanol, ethanol, acetone, and NO_2_ were collected under pseudorandom operation of the μLED. The sampling rate of the sensor signal is 10 Hz, which is well-synchronized to the input signal of the μLED. The dynamic responses (Log_10_(*R*/*R*_0_), where *R* and *R*_0_ are the real-time sensor resistance and the average resistance in air, respectively) and spectrograms of the sensor signals to ethanol and methanol are shown in Fig. 2e, f, respectively. Fig. S6 explains more detailed pre-processing flow and dimensions of sensing data. Here, computed spectrograms originally have 301 spectral components. In order to reduce the size of data and computing load during the deep-learning process, intra-class variance (defined as the variances of spectrum values in the same gas species) and between-class variance (defined as the average values of species-to-species variances) of each spectral component were computed. Then, highly ranked 46 spectral components were selected, which have low intra-class variances and high between-class variances. A low value of intra-class variance and high value of between-class variance mean high reproducibility and good separation for different gas categories^37^. By this method, input data for deep-learning could be lightened. Data for acetone and NO_2_ are provided in Fig. S7. The gray curves in the graph represent raw sensor responses, and the red curves represent moving averages in a 60 s time window. The moving average lines (DC components of the signal) basically follow the general tendency of n-type SMOs exposed to reducing gases. In addition, it is evident that the spectrograms vary depending on the gas environment.

### Deep-learning-based gas identification results

**Fig. 3:**
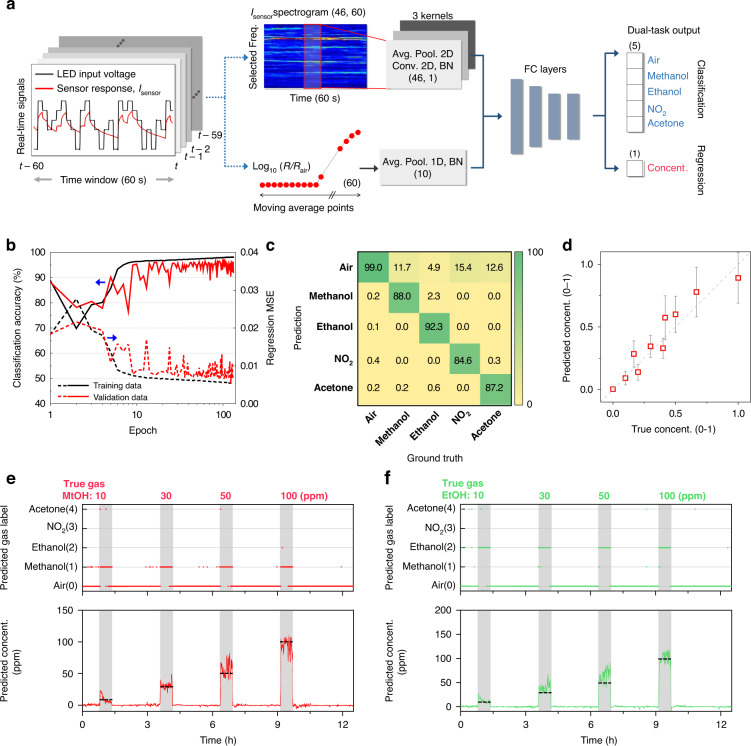
Deep-learning-based identification of mono-gas environment. **a** Architecture of deep convolutional neural network (D-CNN) for classifying five gas species (air, methanol, ethanol, NO_2_, and acetone) and for quantifying the concentrations of each gas. **b** Classification accuracy and regression loss for the training and validation datasets with respect to the training iterations. **c**, **d** Gas species prediction results summarized in a confusion matrix and gas concentrations normalized to 0–1 for test dataset. The predicted gas concentrations are close to the identity line (*y* = *x*) with *r*^2^ = 0.888. **e**, **f** Real-time prediction of gas species and concentrations of methanol and ethanol. Here, normalized gas concentrations are converted to ppm by multiplying the maximum tested concentration of each gas

A deep convolutional neural network (D-CNN) was employed to categorize the type and to quantify the concentration of the target gas from the complex spectrogram data. The D-CNN, with dual-task performance (classification and regression) is designed, as illustrated in Fig. 3a. First, the pseudorandom input and sensor output signals in a unit time window (60 s) are renewed every 1 s (stride interval = 1 s). Thereafter, the frequency spectrum is sequentially calculated. The last calculated spectrum is concatenated with the 59 spectral datasets for the last 60 s that are stored in memory, forming a 2-dimensional spectrogram. In addition to the spectrogram, the DC component of the sensor signal can provide important information regarding the gas concentration, as shown in Fig. 2e, f. Therefore, a sequence of 60 moving average points of the sensor signal is also supplied as an input to the neural network. The spectrogram and moving averages are first processed by the respective average pooling layers and convolution kernels. As the chemical reactions are time-domain phenomena, convolution layers can extract temporal features from the spectrograms more effectively. Tensors processed by the convolutional layers merge and propagate to the fully connected (FC) layers. Each hidden layer is composed of a linear transformation layer (***F***_l+1_ = ***W***_l_ ∙ ***F***_l_ + ***B***_l_), a batch-normalization layer, and a leaky-ReLU (rectified linear unit) activation function (negative slope = 0.01). The output layer is separated into a five-channel classification node and a one-channel regression node. The classification layer, composed of the linear transformation layer and softmax function ($y_k = \exp \left( {a_k} \right)/\mathop {\sum}\nolimits_{{{{\mathrm{i}}}} = 1}^{{{\mathrm{n}}}} {{{{\mathrm{exp}}}}(a_{{{\mathrm{k}}}})}$), outputs the index of the channel with the maximum probability representing the predicted gas species. In contrast, the regression layer performs linear transformation and unit conversion to ppm that estimates the gas concentration. Finally, the real-time outputs are filtered to reduce noises (mode filter and average filter for the classification and regression outputs, respectively). The architecture of the D-CNN is illustrated in detail in Fig. S8.

For training the D-CNN, we collected 624k frames of the spectrogram and the corresponding moving averages for pure air, methanol, ethanol, acetone, and NO_2_. The ratio of the training, validation, and test datasets was set to 4:1:1. All datasets were labeled with ground-truth gas species and concentrations to conduct a supervised learning. The gas concentration (ppm) was normalized by the maximum concentration of each tested gas to reduce the bias resulting from the difference in the tested concentration range for each gas type. The total training loss is defined as $L_{{{{\mathrm{total}}}}} = w \cdot L_{{{{\mathrm{cross}}}} - {{{\mathrm{entropy}}}}} + (1 - w) \cdot L_{{{{\mathrm{MSE}}}}},$ where *L*_cross-entropy_, *L*_MSE_, and *w* are the cross-entropy loss ($L_{{{{\mathrm{cross}}}} - {{{\mathrm{entropy}}}}} = - \mathop {\sum}\nolimits_{{{\mathrm{i}}}}^{{{\mathrm{N}}}} {t_{{{\mathrm{i}}}}{{{\mathrm{log}}}}(y_{{{\mathrm{i}}}})}$) of the classification outputs, mean-square error (MSE, $L_{{{{\mathrm{MSE}}}}} = \frac{1}{2}\mathop {\sum}\nolimits_{{{\mathrm{i}}}}^{{{\mathrm{N}}}} {(y_{{{\mathrm{i}}}} - t_{{{\mathrm{i}}}})^2}$) of the regression outputs, and weighting factor, respectively. The weighting factor (0 < *w* < 1) is a kind of hyperparameter to roughly match the scales of two loss functions and allow the training to proceed evenly for the classification and regression. Here, the constant weighting factor of 0.4 was used during the training progress. The Adam optimizer with a constant learning rate (*η* = 0.001*)* was used to minimize the total training loss. As shown in Fig. 3b, the D-CNN was trained through 130 epochs without much divergence. The classification accuracy and regression MSE for the validation dataset reached 96.38% and 7.8 × 10^-2^, respectively (Note that the normalized concentrations range from 0 to 1.), at the last iteration. Figure 3c shows the predicted gas species summarized in the confusion matrix for the test dataset. The D-CNN presents accurate prediction of each gases over 85% accuracy. Falsely predicted cases are mostly reactive gases to pure air (methanol to air (11.7%), ethanol to air (4.9%), NO_2_ to air (15.4%), and acetone to air (12.6%)). Overall accuracy was calculated to be 96.99%. By the analysis of result, it has been confirmed that the falsely predicted cases occur mostly in the regions where there is a drastic gas transition during real-time gas prediction.

Figure 3d shows the scatter plot between the normalized true concentration and the predicted concentration for all tested gases. The predicted gas concentrations show high correctness to true values and are close to the identity line (*y* = *x*) with *r*^2^ = 0.888. Figure 3e, f shows the real-time prediction of gas species and concentrations to the methanol and ethanol gases, respectively, by the forward-propagation of time-sequential test datasets into the trained D-CNN. Real-time predictions for NO_2_ and acetone gases are given in Fig. S9. The maximum latency of the prediction is 80 s and 79 s in the response and recovery stages, respectively; therefore, it can be confirmed that the prediction of both the gas species and concentration is sufficiently fast for general applications of real-time gas detection, and the pseudorandom operation of μLED and the processing sensor responses using a D-CNN can effectively identify mono-gas environments.

### Prediction results of gas mixture

Quantitative identification of individual gas species in a gas mixture is the ultimate goal of e-nose technology. Several studies have demonstrated the possibility of identifying gas mixtures using multi-sensor arrays^39–41^. However, multi-sensor-based approaches still lack efficiency in terms of cost, space, and power consumption. Furthermore, real-time identification of gas mixtures has rarely been explored owing to the lack of decoding power in the conventional ML methods. This study demonstrates real-time quantitative identification of a binary gas mixture (specifically methanol and ethanol) using the proposed sensing strategy and deep-learning algorithm. First, the aforementioned dual-task D-CNN has been modified to ensure its suitability for analyzing the mixed gas. The pre-processing method and overall architecture of the D-CNN is maintained as is, while only the output layer is modified. In the modified output layer, the tensors represent the normalized concentrations of the constituent gases and their confidence scores. The binary confidence score is defined by the existence of a specific gas, 1 and 0 indicating the presence and absence of the gas, respectively. Therefore, the dataset must be labeled with true normalized concentrations (0–1) of the constituent gases and their existence (0 or 1). Here, the combination of gas species to be analyzed is expandable by extending the length of the output tensor and pre-training in the appropriate target environments.

**Fig. 4:**
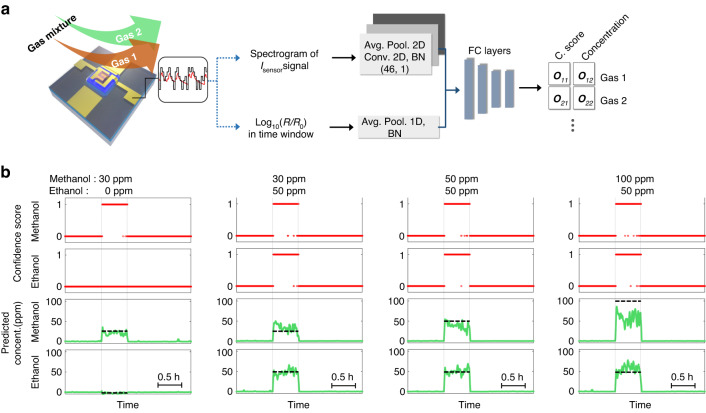
Identification of binary gas mixture. **a** Architecture of modified deep convolutional neural network for identifying gas mixtures. The network output is the normalized concentration of each gas and its confidence score (C. score). The length of the output tensor is expandable according to the appropriate target environments. **b** Real-time identification of gas mixture with various mixing ratios of methanol (0, 30, 50, 100 ppm) and ethanol (0, 30, 50, 100 ppm). The binary C. score (0 and 1) indicates the existence of a specific gas, 1 and 0 representing the presence and absence of the gas, respectively. The result shows accurate identification of gas mixture as well as estimation of gas concentrations of both methanol and ethanol

In order to prove the feasibility of the concept, the sensing data of the various mixture combinations of methanol (0–100 ppm) and ethanol (0–100 ppm) gases were collected under the pseudorandom operation of the μLED. Fig. S10 shows the dynamic responses (Log_10_(*R*/*R*_0_)) and their preprocessed spectrograms with various mixing ratios of methanol and ethanol. The variations of response signals tend to be proportional to the total amount of gas components, and the spectrograms are evidently different owing to the various mixture states. To analyze the features of the spectrograms, the D-CNN was trained with 420k frames of the dataset. The total training loss is defined as $L_{{{{\mathrm{total}}}}} = w \cdot L_{{{{\mathrm{MSE}}}},{{{\mathrm{confidence}}}}} + (1 - w) \cdot L_{{{{\mathrm{MSE}}}},{{{\mathrm{concentration}}}}},$ where *L*_MSE,confidence_, *L*_MSE,concentration_, and *w* denote the MSE of the confidence score, regression outputs, and the weighting factor, respectively. The weighting factor (0 < *w* < 1) is also for matching the scales of two MSEs during the training progress (*w* = 0.4). After 130 epochs of training, the total loss for the validation dataset was 1.2 × 10^-2^, without any overfitting, as shown in Fig. S11. Figure 4 shows the real-time identification of gas mixtures with the various mixture ratios of methanol and ethanol gases. Confidence scores clearly indicate the presence of gas in both the mono-gas environment (30 ppm methanol and 0 ppm ethanol) and the gas mixture environment (30 ppm methanol and 50 ppm ethanol; 50 ppm methanol and 50 ppm ethanol; 100 ppm methanol and 50 ppm ethanol). The overall accuracy of the confidence scores, evaluated by a threshold criterion (confidence score <0.5 as 0 (no gas) and confidence score ≥0.5 as 1), are 97.63% and 98.68% for methanol and ethanol, respectively. Further, the predicted concentrations of the gases show adequate accuracy when compared with the true value. Mean absolute percentage error (MAPE) of the predicted concentrations are 36.8% and 32.3% for methanol and ethanol in the non-mixing (i.e. mono-gas) state, respectively, as summarized in Fig. S12. Furthermore, the modified D-CNN successfully estimates the concentration of each gas constituent in the mixtures (MAPEs of predicted concentrations are 34.5% and 34.3% for methanol and ethanol, respectively). The results of various mixture ratios of methanol and ethanol are provided in Fig. S13.

## Discussion

In Table 1, this study is compared with some recent studies pertaining to ML-based e-nose technology. Whereas most conventional studies utilized 8–20 multi-sensor array for gas identification^22–24^, the current study uses only a single sensor, providing a much more efficient approach for the gas identification in terms of cost, space, and power consumption. Although there have been other studies to reduce the power consumption using a single sensor, global heating was still used and did not show actual reduction of the power consumption^25,42,43^. Particularly, the μLP gas sensor used in this study consumes merely 526 μW, which is a record low power consumption among the various activation sources for the SMO-based e-nose technology. State-of-the-art microheater-based gas sensors have achieved sub-10 mW power comsumption^44–46^, and further power-saving has necessitated high-cost nanofabrication techniques^47,48^. On the other hand, the μLP gas sensors that are based on cost-effective micro-fabrication processes facilitate microwatt-level average power consumption. Additionally, previous monolithic LED gas sensors could cause electrical shorts due to the sensing materials^34,49^. In this study, double SiO_2_ insulating layers were used to prevent electrical shorts between the sensor electrode and the *p*–*n* electrodes of μLED. Therefore, the proposed sensing device can greatly improve the efficiency of the e-nose system and has a great potential for using any gas-sensing material without electrical shorts. Some carbon nanomaterial-based sensors in Table 1 consume negligible electrical power, owing to their operation at room temperature and no requirement of activation sources^23,42^. However, no gas quantification capabilities were demonstrated for theses carbon nanomaterial-based sensors, possibly due to the limited reversibility, linearity, and speed of the sensor response. With respect to the analysis method, the proposed D-CNN exhibits highly accurate gas-identification performance in both the classification task (accuracy = 96.99%) and regression task (MAPE = 31.99%), when compared with the conventional ML analysis methods, such as multilayer perceptron (MLP), k-nearest neighbors (KNN), and support vector machine (SVM). In particular, this study exhibits real-time identification capability for the entire monitoring period, whereas most conventional e-nose studies only demonstrated the performance in the steady-state sensor responses, excluding transient responses in the drastic gas transition periods. Furthermore, the proposed sensing strategy facilitates the accurate analysis of gas mixtures and highly improves its utility in the complex gas environments of real fields. Although there is a study that has conducted classification of each gas in the gas mixtures (CO and CH_4_) with a commercial SMO gas sensor array, there has been no study that quantified the concentration of each gas in real-time^50^. Figure S14 summarizes the advantages of the single sensor-based electronic nose system developed in this study using the μLP gas sensor and pseudorandom illumination. In addition, although the highly sensitive gold NPs coated GLAD In_2_O_3_ has been utilized to demonstrate the proof-of-concept of the research in this study, the suggested time-variant light illumination method is expected to work universally for various sensing materials. Therefore, exploring new sensing materials will be continued for future studies.

**Table 1:** Summary of machine-learning-based electronic nose technology-related studies in the last three years

	Liu et al.^22^	Schroeder et al.^23^	Thai et al.^24^	Tonezzer et al.^25^	Hayasaka et al.^42^	Zhang et al.^50^	Kanaparthi et al.^43^	Present study
Sensor type	SMO (commercial)	SWCNT	SMO + microheater	SMO + global heating	Graphene FET	SMO (commercial)	SMO + global heating	SMO + micro-LED
No. of sensors	10	20	8	1	1	6	1	1
No. of test gases	6	5	5	7	3	2	3	4
Total sensor power	30 W (datasheet)	NQ	340 mW (170 × 2)	NC	NQ	3.2 W (datasheet)	NQ	526 μW
Analysis method	BPNN	KNN	SVM	PCA, SVM	MLP	BPNN	RF	D-CNN
Classification accuracy (%)	99.8	91	100	94.3	99.6	93.29	99.8	96.53
Regression error	0.7919 (RMSE)	NC	8-28% (MAPE)	18.4% (MAPE)	NC	NQ	NC	31.99 % (MAPE)
Real-time prediction	No	No	No	No	No	No	No	Yes
Multi-gas mixture	No	No	No	No	No	Yes	No	Yes

*SMO* semiconductor metal oxides, *SWCNT* single-walled carbon nanotube, *FET* field-effect transistor, *LED* light-emitting diode, *BPNN* back-propagation neural network, *KNN* k-nearest neighbors, *PCA* principal component analysis, *SVM* support vector machine, *MLP* multilayer perceptron, *D-CNN* deep convolutional neural network, *RMSE* root mean-square error, *MAPE* mean absolute percentage error, *NC* not considered, *NQ* not quantified

In summary, this paper proposed a novel sensing strategy to identify gas species selectively and to estimate the concentrations of multiple gases. The proposed method applies pseudorandom input to a single monolithic μLP gas sensor. Transient sensor signals, owing to the rapid changes in the light intensity of μLED and different reaction kinetics of various gas species, facilitate the identification of gas species. In this study, the excellent durability of the GaN-based μLED facilitates drastic, wide-range alternation in the light intensity and long-term use. A D-CNN was used to effectively analyze the complex frequency spectrogram of the transient sensor signals. As such, the identification of four mono-gas environments and binary gas mixtures of the two selected gases (ethanol and methanol) were successfully demonstrated with high accuracy. The proposed method, using a single chemoresistive gas sensor, is expected to provide the most efficient method for analyzing various gas environments in terms of cost, space, and power consumption. Therefore, the proposed concept is expected to be used extensively in real-life applications in environmental monitoring, disease diagnosis, food process monitoring, and agricultural fields. For future research, further collection and investigation of the big data corresponding to gas-sensing in various environments will be continued. For this, system-level integration of sensors, analog-front-end circuits, processors with capabilities for computing the lightweight ML will be necessary. For example, the device-edge ML computation with a neuromorphic processor will allow more rapid, power-efficient gas identification in the actual application fields. In addition, further studies of sensing materials with improved optical and sensing properties will be investigated.

## Materials and methods

### Fabrication of μLED-embedded photoactivated (μLP) gas sensors

The fabrication process of the μLP gas-sensing platform is based on the method proposed in our previous work^34^. In this study, the indium concentration in the InGaN/GaN MQW layers was precisely tuned for the near-UV emission (approximate emission wavelength of 395 nm) during a MOCVD process. The emission area was designed to be 50 × 50 μm^2^. After fabrication of μLP gas sensors in wafer-scale, they were diced into a single sensor chip size of 5 × 5 mm^2^ through a blade dicing. Prior to integrating the sensing material, a 3.5 μm-thick photoresist (AZ nlof 2035, MicroChemicals, Germany) was patterned on the pre-fabricated μLP platform to define the sensing area. Nano-porous In_2_O_3_ films were deposited based on the GLAD method using a radio frequency (RF) sputtering system. The specific process conditions were as follows: argon atmosphere with a pressure of 4 mTorr, tilt angle of 85°, rotation speed of 3.6 rpm, RF power of 250 W, and a deposition time of 90 min. Next, gold NPs were coated on the GLAD In_2_O_3_ surface by e-beam evaporation with a deposition thickness of 1 nm measured by QCM sensor. Thereafter, the GLAD In_2_O_3_ film coated with gold NPs was patterned by a lift-off process in acetone.

### Characterization of the fabricated devices

The fabricated sensor devices were observed using field emission scanning electron microscopy (FE-SEM; SU8230, Hitachi, Japan). The porosity of the deposited In_2_O_3_ sensing layer was approximated through an image processing of the top-view SEM image, as shown in Fig. S3. The porosity (*φ*) is generally defined as *φ* = (*volume of voids*)/(*total volume*) × 100 (%) ≈ (*top-view area of voids*)/(*total top-view area*) × 100 (%), assuming that the film has a prismatic columnar structure. The optical and electrical properties of the μLEDs were obtained using an L-I-V measurement system (OPI 160, WITHLIGHT, S. Korea) with an integrating sphere and a source-meter (Keithley 2400, USA).

### Data acquisition

The sensor devices were mounted in a customized testing chamber and connected to a dual-channel source-meter (Keithley 2636b, USA) to apply a forward bias to the μLED and measure the sensing resistance at a sampling rate of 10 Hz. The pseudorandom input signal, composed of five levels of voltages (2.2, 2.6, 2.8, 3.0, and 3.2 V) with a fixed time interval (2 s) for each level, was generated by an interface software (LabVIEW, National Instruments, USA). Gas was supplied to the sensor device, and the concentration of the tested gases (methanol, ethanol, NO_2_, and acetone) was monitored by controlling the flow rates of each gas and dry air with mass flow controllers (MFC). More details are summarized in Fig. S15.

### Deep-learning-based gas identification

The open source machine-learning library (PyTorch, Meta, USA) was utilized to construct the deep convolutional neural network (D-CNN). Training of the D-CNN was accelerated using a high-performance GPU (RTX Titan, NVIDIA, USA)-based computing environment. The architecture of the D-CNN is illustrated in detail in Fig. S8. For the best training results, hyperparameters of D-CNN learning was tuned, such as the size of the network, activation/loss functions, the learning rate for Adam optimizer, and weightings for classification and regression losses.

## Supplementary information

Supporting Information

Supplementary_video

## Data Availability

The data that support the plots within this paper and the other findings of this study are available from the corresponding authors upon reasonable request.
