# Correction: Deep-learning-based gas identification by time-variant illumination of a single micro-LED-embedded gas sensor

**DOI:** 10.1038/s41377-025-02023-5

**Published:** 2025-11-11

**Authors:** Incheol Cho, Kichul Lee, Young Chul Sim, Jae-Seok Jeong, Minkyu Cho, Heechan Jung, Mingu Kang, Yong-Hoon Cho, Seung Chul Ha, Kuk-Jin Yoon, Inkyu Park

**Affiliations:** 1 Department of Mechanical Engineering, Korea Advanced Institute of Science and Technology (KAIST), 291 Daehak-ro, Yuseong-gu, Daejeon, 34141 Republic of Korea; 2 Department of Physics, Korea Advanced Institute of Science and Technology (KAIST), 291 Daehak-ro, Yuseong-gu, Daejeon, 34141 Republic of Korea; 3 SENKO Co., Ltd., 485, Oesammi-Dong, Osan-Si, Gyeonggil-Do 18111 Republic of Korea

**Keywords:** Lasers, LEDs and light sources, Electronics, photonics and device physics

Correction to: *Light: Science & Applications* 10.1038/s41377-023-01120-7

published online 18 April 2023

Following publication of the original article, an error was identified in Figure 4b. The correct figure 4 is shown below:
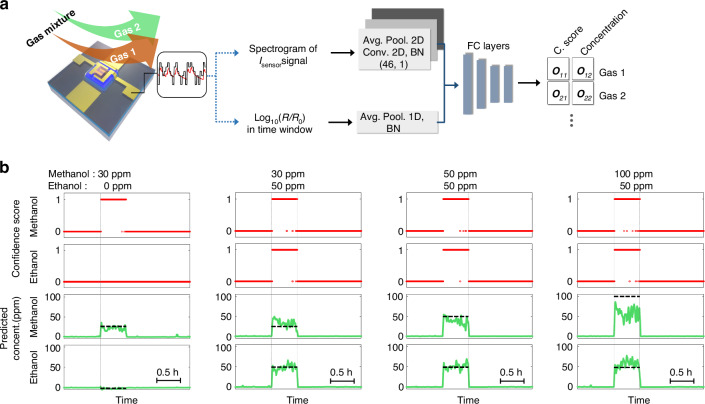


The original paper has been updated.

