# Supplementary material for: Deep-learning-based gas identification by time-variant illumination of a single micro-LED-embedded gas sensor
Source: Light Sci Appl. 2023 Apr 18;12:95. doi: 10.1038/s41377-023-01120-7 (PMC10113244; doi:10.1038/s41377-023-01120-7)
Supplement: Supplementary file 1 — Supporting Information [file 41377_2023_1120_MOESM1_ESM.pdf]

Supplementary Information for

Deep-Learning-based gas identification by time-variant illumination of a single micro LED-embedded gas sensor

Incheol Cho[1], Kichul Lee[1], Young Chul Sim[2], Jae-Seok Jeong[1], Minkyu Cho[1], Heechan Jung[1], Mingu Kang[1], Yong-Hoon Cho[2], Seung Chul Ha[3], Kuk-Jin Yoon[1,], Inkyu Park[1,*]*

[1] Department of Mechanical Engineering, Korea Advanced Institute of Science and Technology (KAIST), 291 Daehak-ro, Yuseong-gu, Daejeon, 34141, Republic of Korea

[2] Department of Physics, Korea Advanced Institute of Science and Technology (KAIST), 291 Daehak-ro, Yuseong-gu, Daejeon, 34141, Republic of Korea

[3] SENKO Co., Ltd., 485, Oesammi-Dong, Osan-Si, Gyeonggil-Do 18111, Republic of Korea

Corresponding authors: Inkyu Park (inkyu@kaist.ac.kr), Kuk-Jin Yoon (kjyoon@kaist.ac.kr),

Supplementary figures.

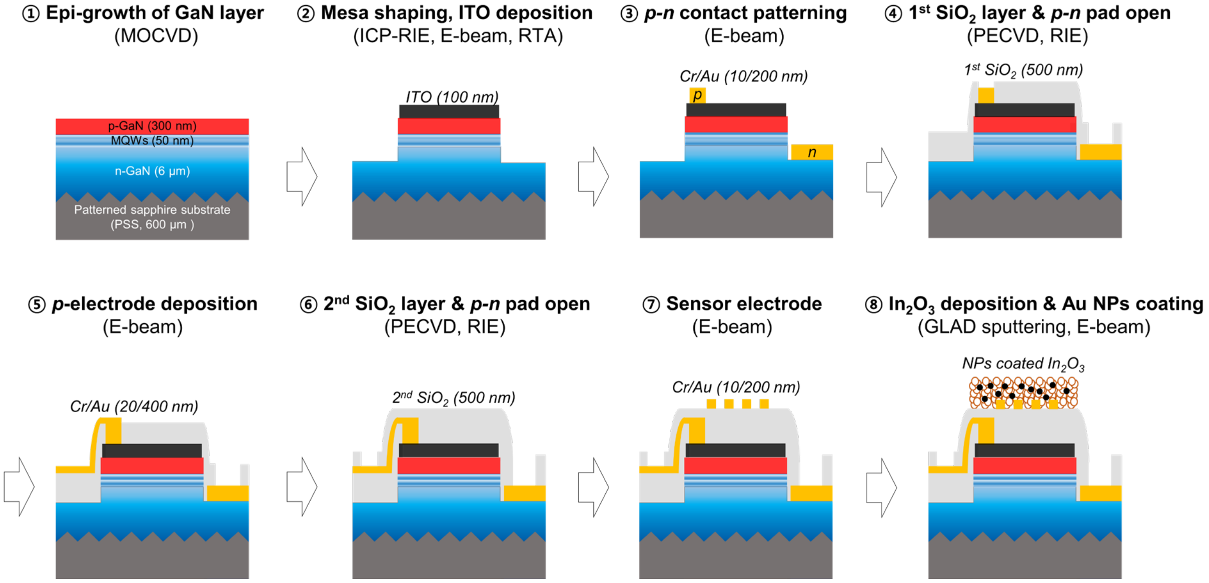

Figure S1. Fabrication process of a µLED-embedded photoactivated gas sensor. A stack of *n*-type GaN, InGaN/GaN multi-quantum well (MQW) layer, and *p*-type GaN layer was epitaxially grown on a sapphire substrate through a metal-organic chemical vapor deposition (MOCVD) (step ①). GaN layers were vertically etched via inductively coupled plasma-reactive ion etching (ICP-RIE) to formulate mesa structures of µLEDs (step ②). Silicon dioxide (SiO_2) insulation layer was deposited by plasma-enhanced chemical vapor deposition (PECVD) process (step ④ and ⑥). PECVD SiO_2 were vertically etched via reactive ion etching (RIE) to open up the electrical connection area(step ④ and ⑥). ITO and Au/Cr contact electrodes were patterned by UV photolithography and e-beam evaporation (step ②, ③, ⑤, and ⑦). Nano-porous indium oxide (In_2O_3) film was deposited through the glancing angle deposition (GLAD) method with a RF sputtering system and gold nanoparticles were coated on In_2O_3 through e-beam evaporation (step ⑧).

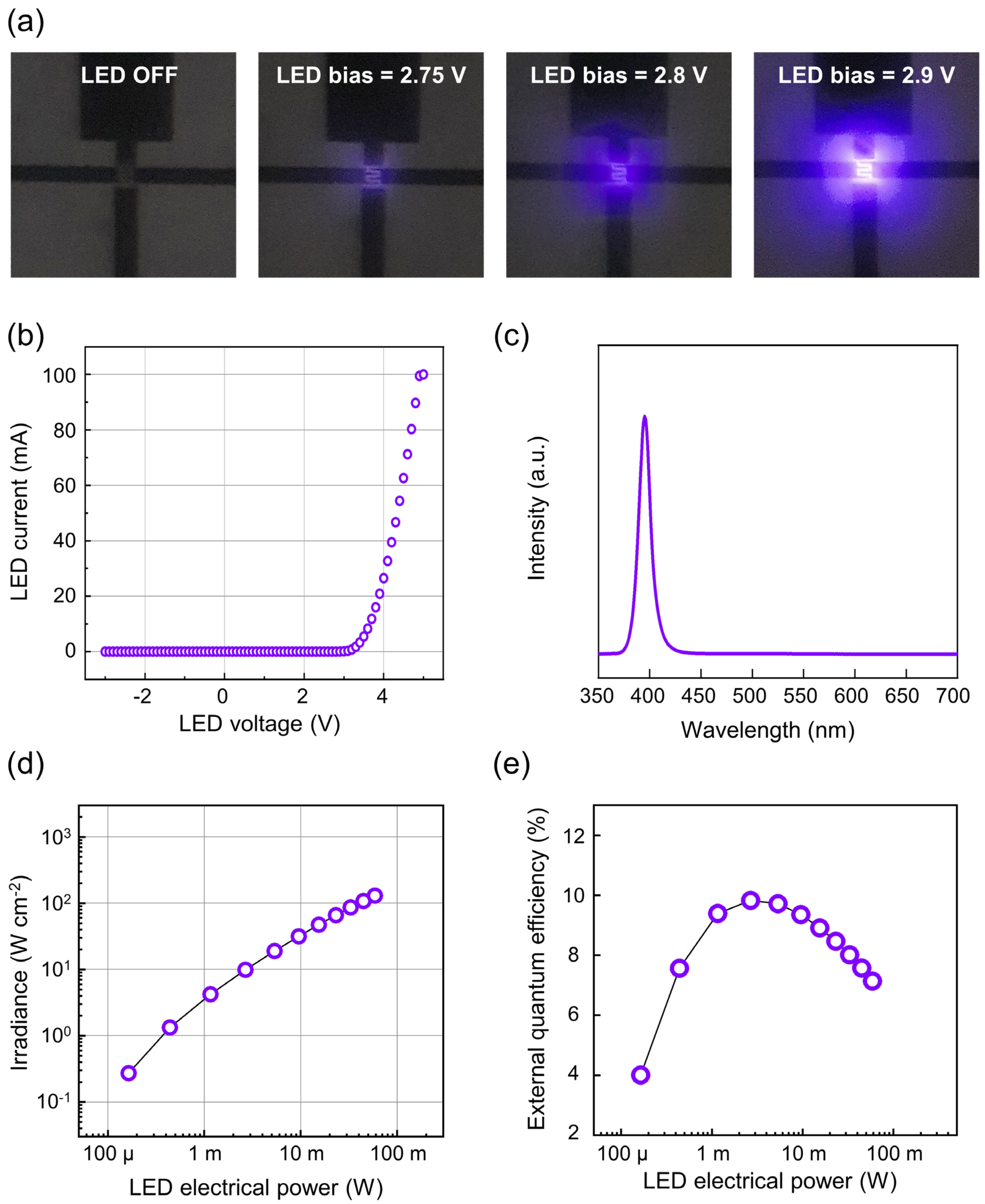

Figure S2. L-I-V Characterization of the fabricated μLED. (a) Photomicrograph of light-emitting micro-LEDs according to different LED voltages. (b) I-V curve. (c) Light emission spectrum. (d) Irradiance depending on the input electrical power. (e) External quantum efficiency depending on the input electrical power.

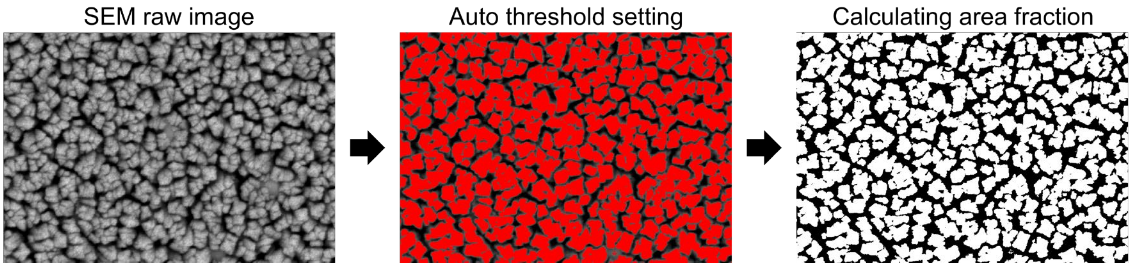

Figure S3. The flow of image processing to capture the area fraction of voids and cracks from the top-view SEM image of the columnar In_2O_3 film.

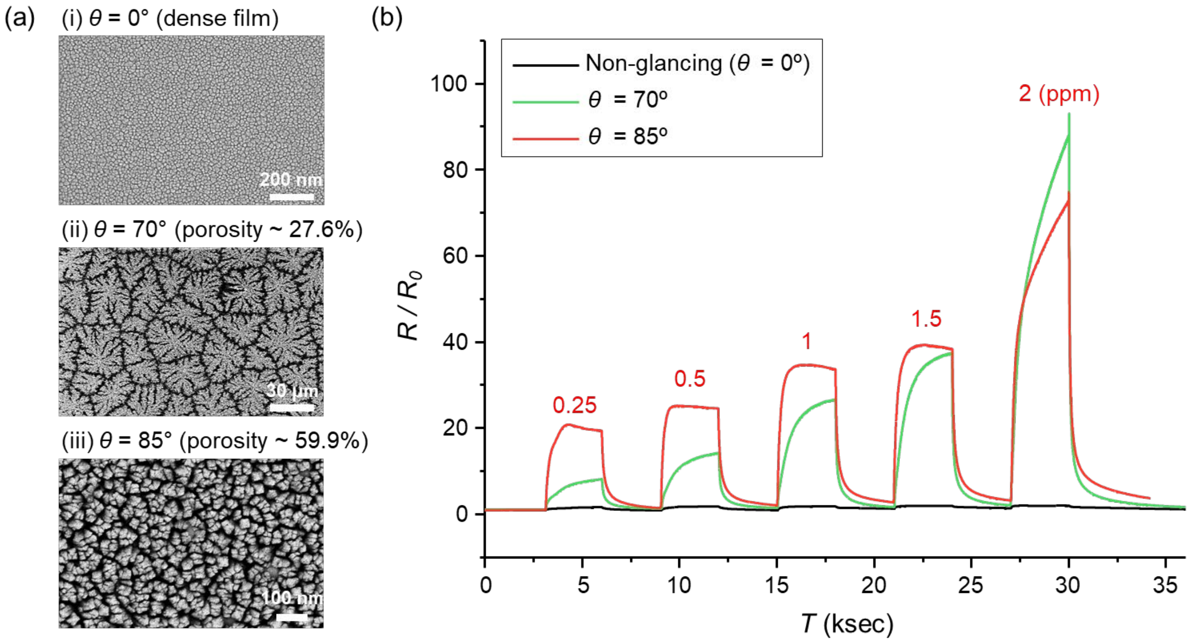

Figure S4. (a) SEM images and (b) dynamic sensor responses of porosity-controlled In_2O_3 (tilting angle (θ) = 0, 70, and 85°). Gas tests were conducted with NO_2 gas under the same light illuminating condition (peak wavelength = 395 nm).

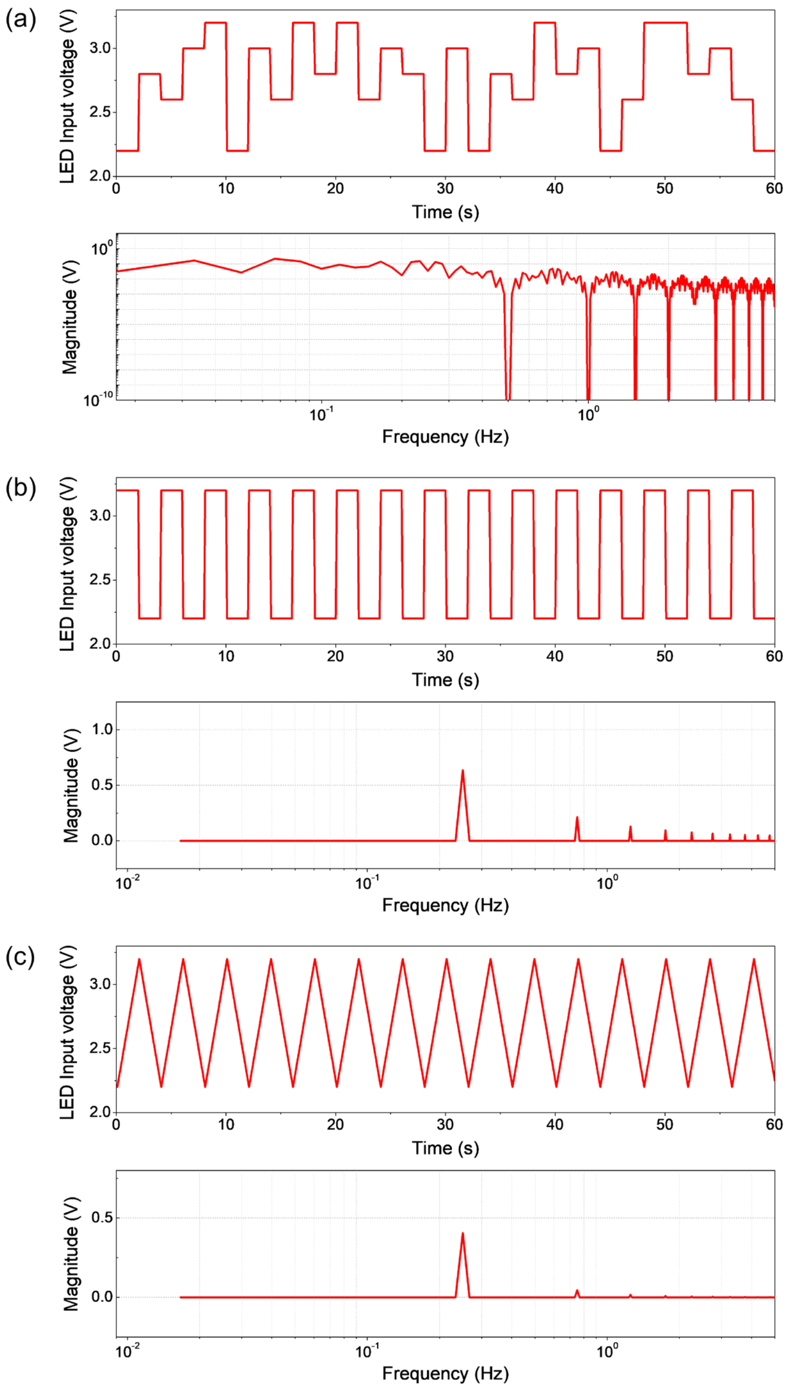

Figure S5. Various types of LED input signals and their frequency spectra. (a) Pseudorandom signal. (b) Simple square wave signal. (c) Cyclic ramp-up and down signal. As random signals contain all frequency components like white noise, it is not needed to investigate the most advantageous operating frequency for gas discrimination in advance.

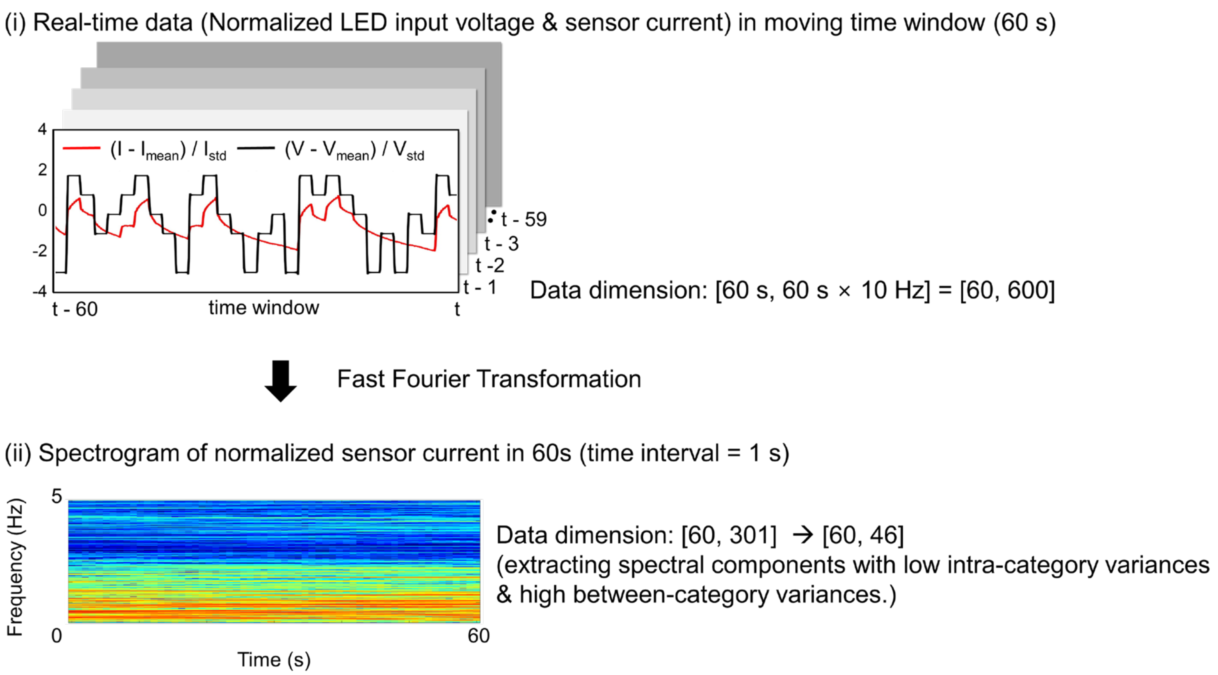

Figure S6. Description of a preprocessing method and dimensions of tensor data. Firstly, the sensor and pseudorandom input signals in a unit time window (60 sec) are renewed every 1 sec (stride interval = 1 sec). Then, frequency spectrum of the sensor current is sequentially calculated. The lastly calculated spectrum is concatenated with the 59 spectra data for the last 60 sec that are stored in memory, forming a 2-dimensional spectrogram.

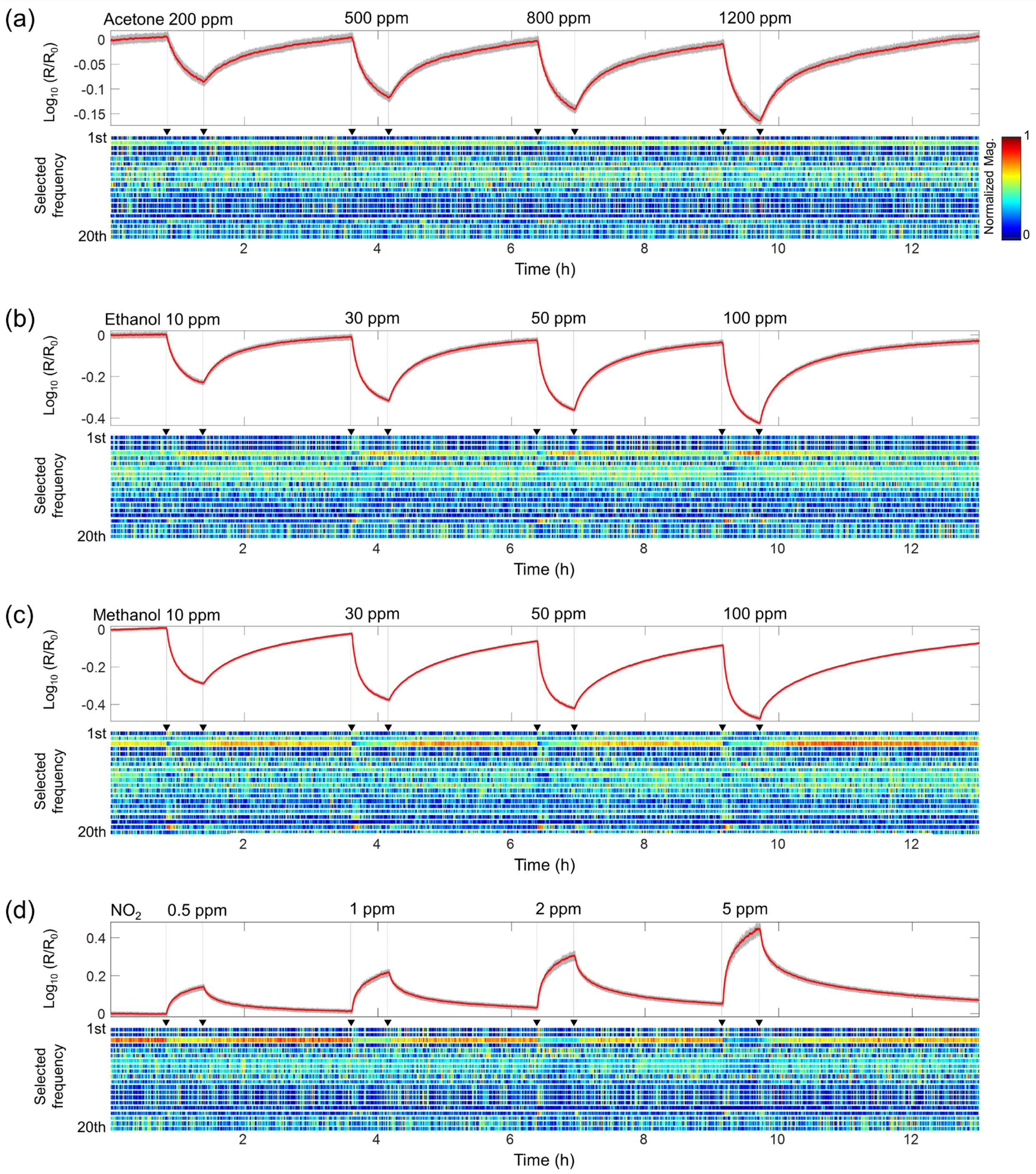

Figure S7. Results of gas tests under the pseudorandom operation. Dynamic responses ($Log_{10}(R/R_0)$) and spectrograms of sensor signals to acetone, ethanol, methanol, and NO_2 with various concentrations. Gray lines are raw transient signals and red lines are moving averages in a 60 sec time window. The highly ranked 46 spectral components were used for the spectrogram data, but only 20 components are shown in the figure.

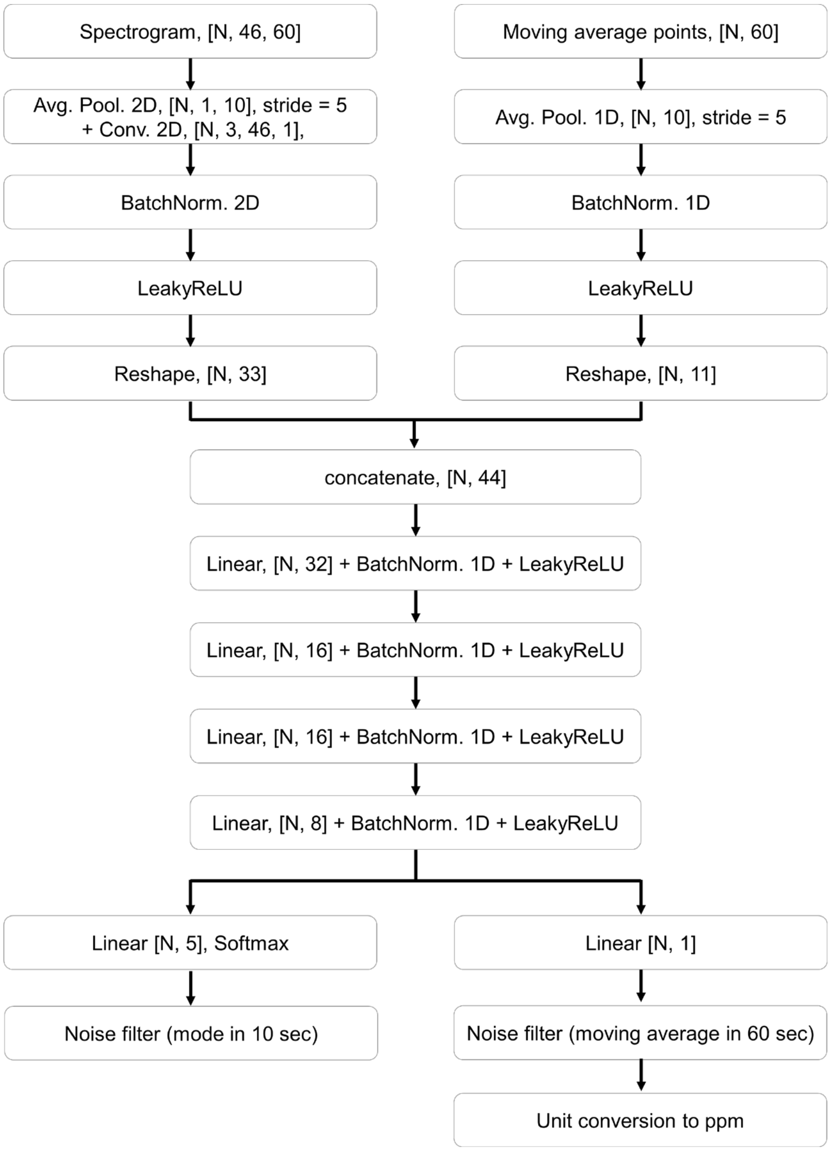

Figure S8. Full architecture of the dual-task deep convolutional neural network (D-CNN) for gas identification. N is the size of batch.

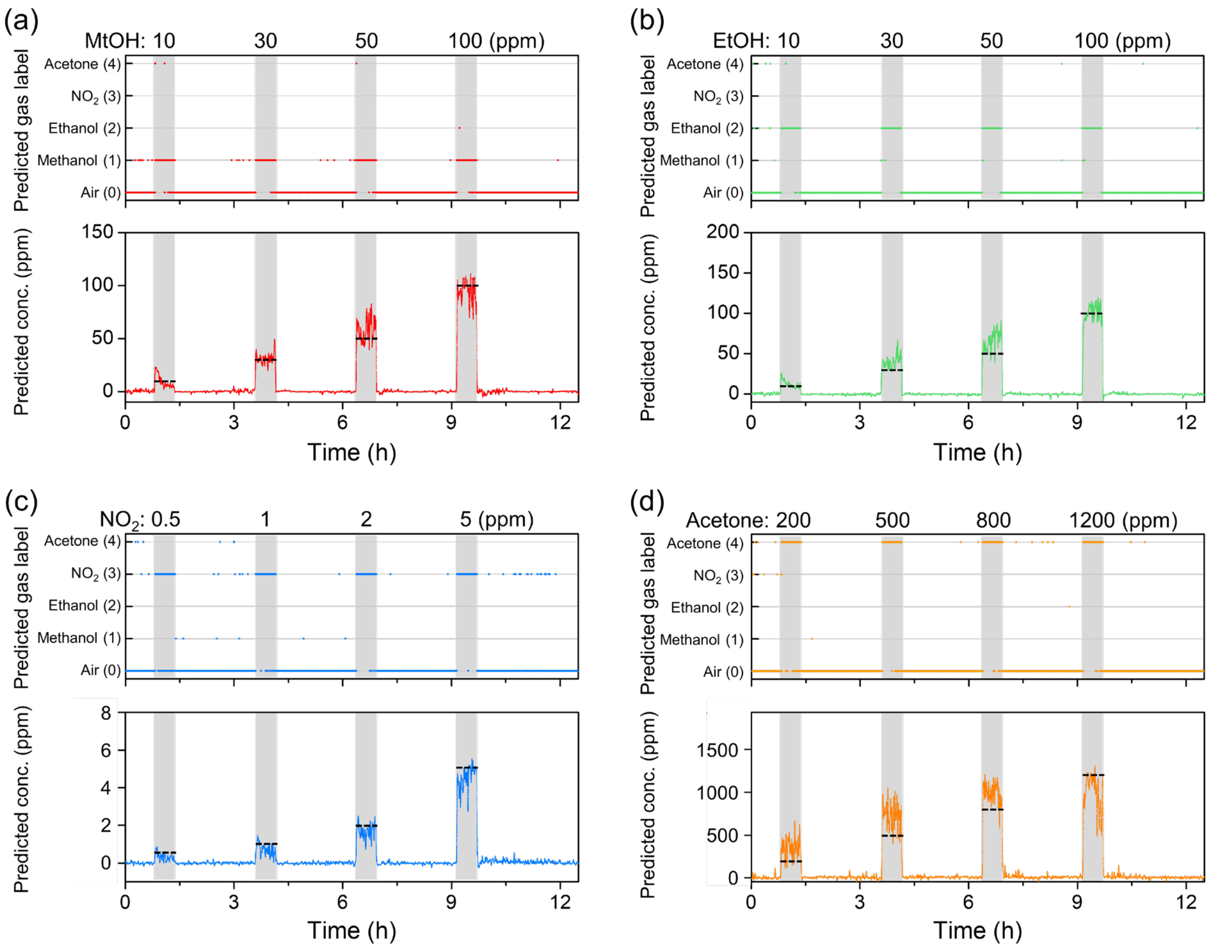

Figure S9. Real-time prediction of gas species and concentrations to the methanol, ethanol, NO_2, and acetone.

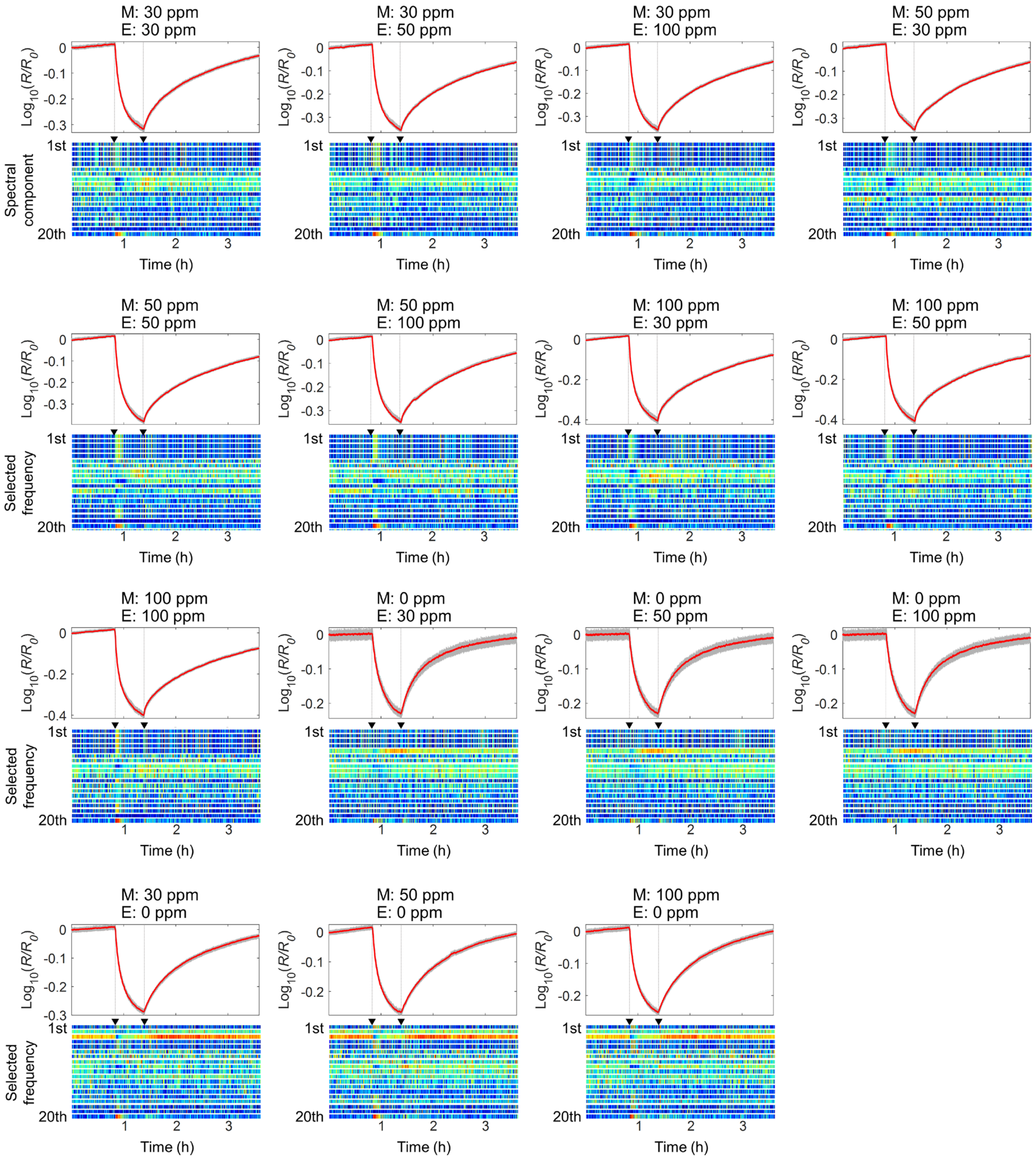

Figure S10. Collected dynamic responses ($\mathrm{Log}_{10}(R/R_0)$) and spectrograms to gas-mixture with various mixing ratios of methanol (M) and ethanol (E). Gray curves are raw transient signals and red curves are moving averages in a 60 sec time window.

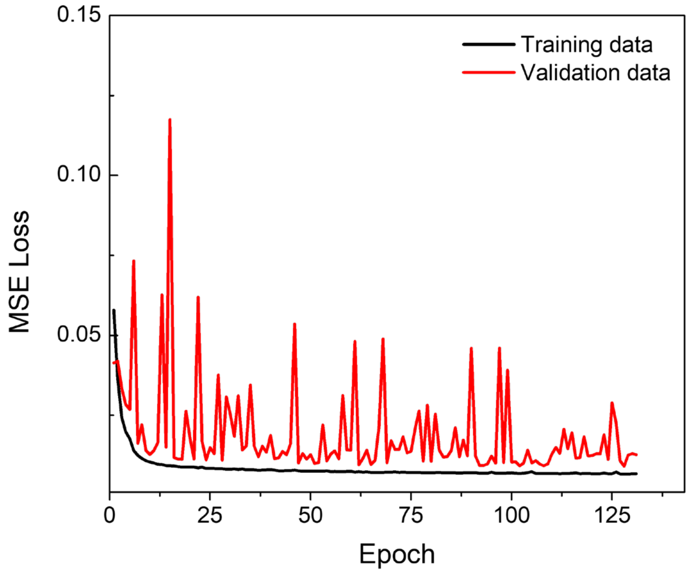

Figure S11. Regression loss of modified D-CNN for the training and validation datasets of gas mixture situation with respect to the training iterations.

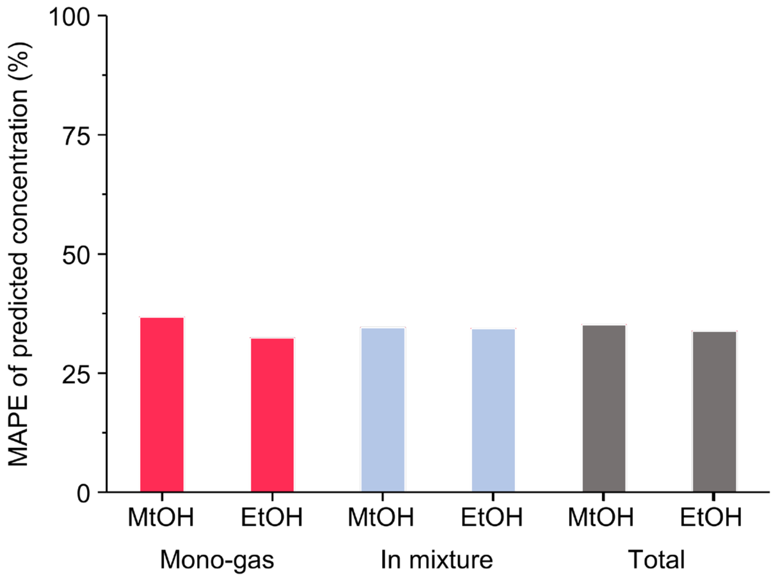

Figure S12. Summarized mean absolute percentage errors (MAPEs) of predicted concentration to the non-mixing and mixing states.

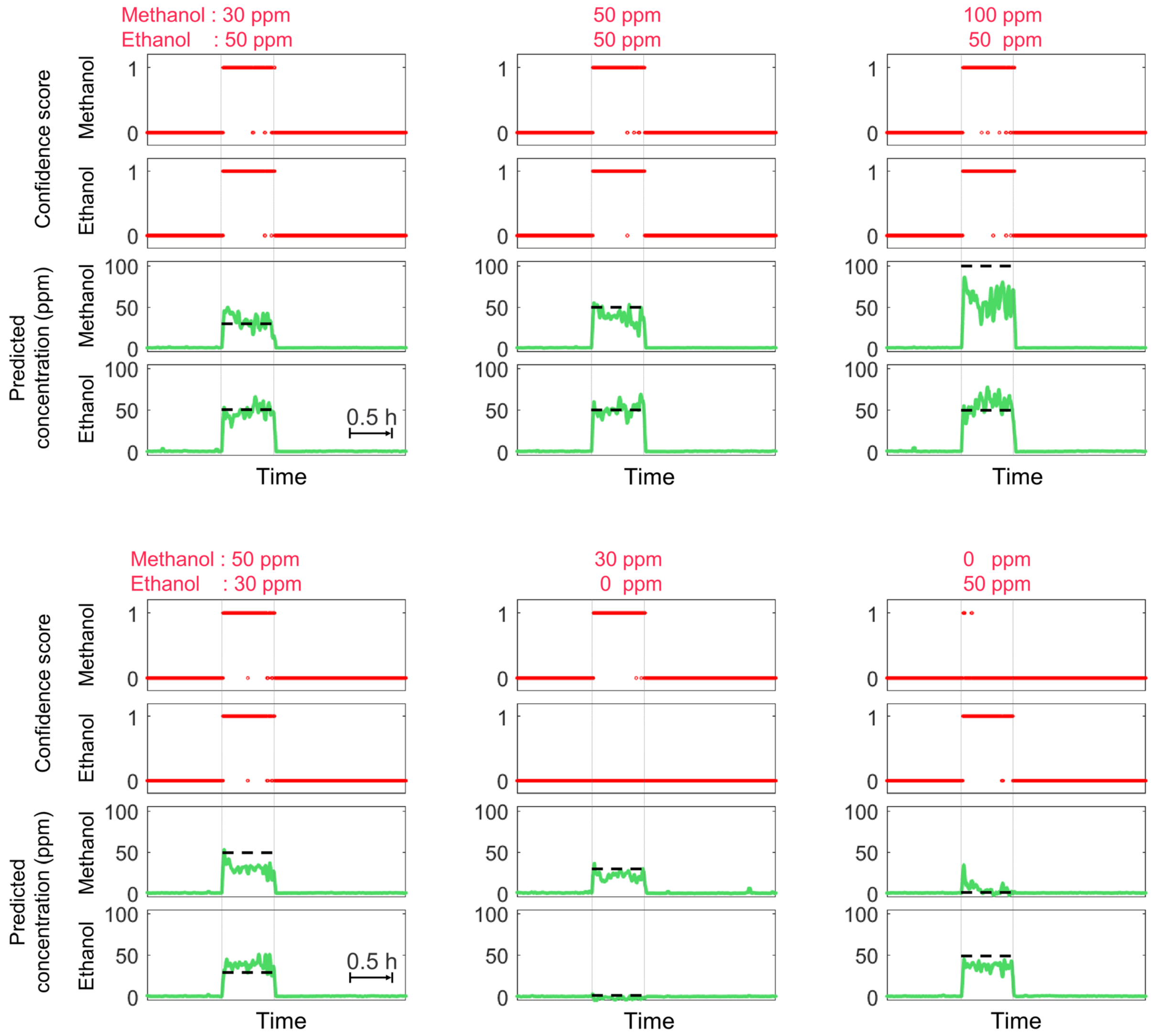

Figure S13. Real-time identification of gas-mixture with various mixing ratios of methanol and ethanol.

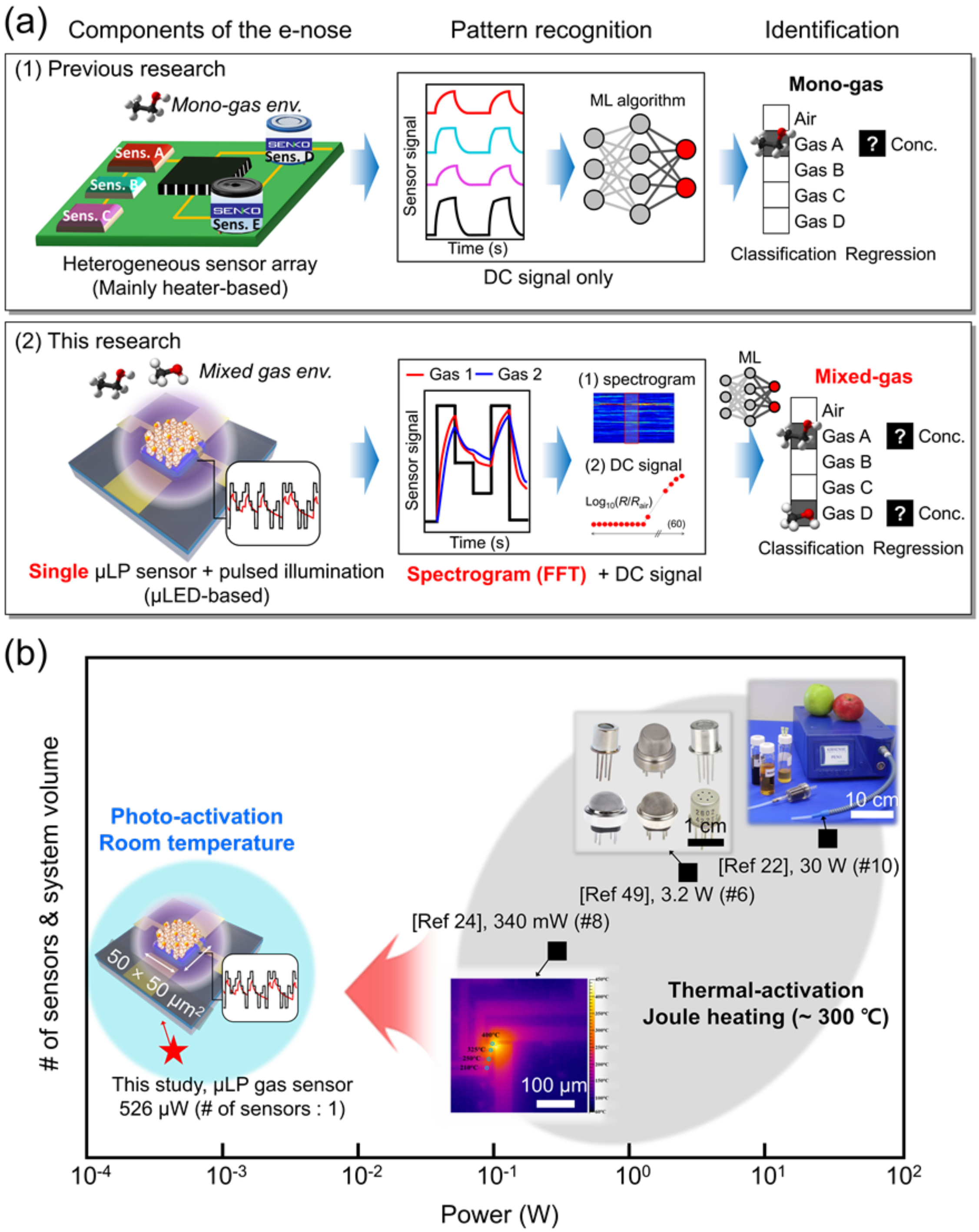

Figure S14. (a) The difference between the previously developed e-nose systems and the µLP-based e-nose system developed in this study. Most e-nose systems consisted of multiple gas sensors based on thermal activation using an embedded heater. (b) Efficiency of µLP-based e-nose compared to conventional heater-based e-nose systems. Total power consumption has been reduced to less than one-hundredth those of thermally activated e-nose systems.

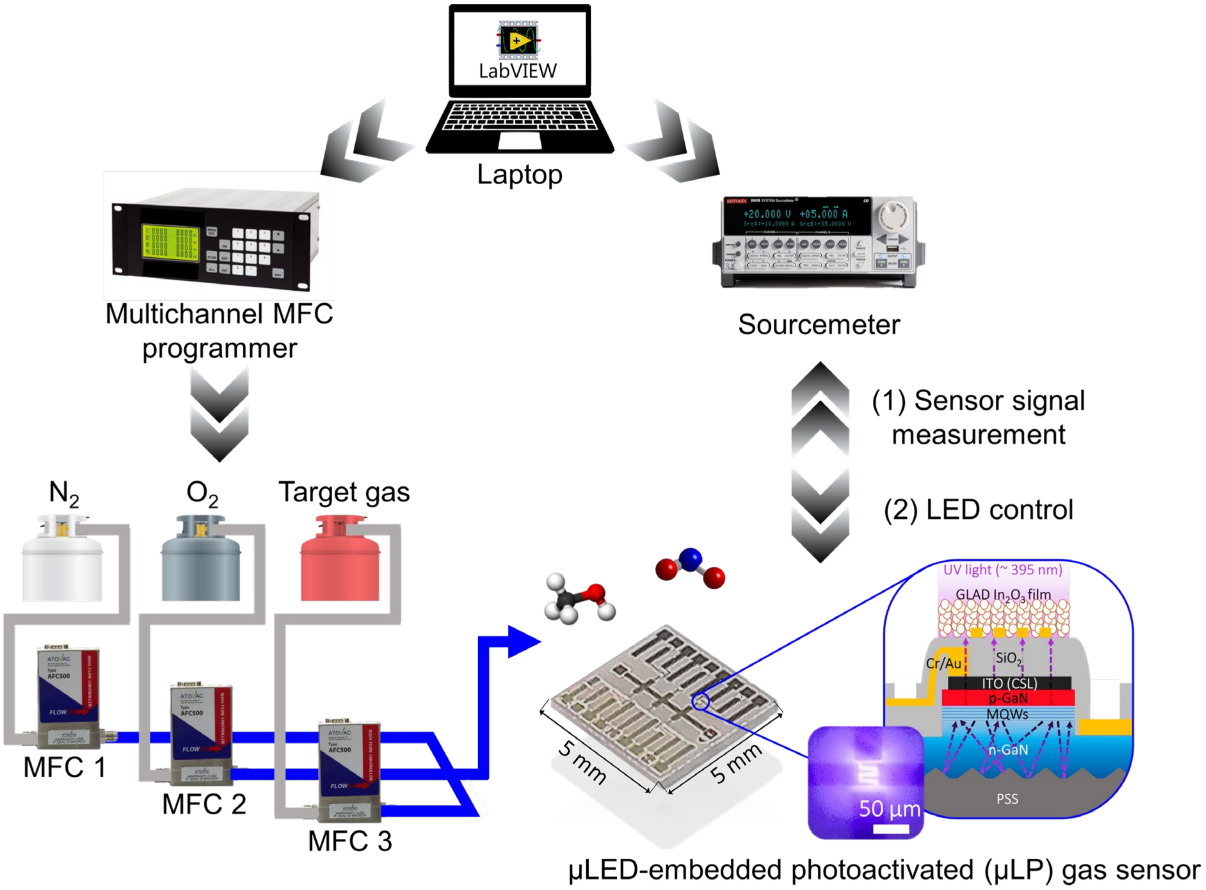

Figure S15. Gas-sensing setup used in this research. A sourcemeter was used for the sensor signal measurement and µLP control. MFC allows to input an accurate flow rate of each gas to the gas test chamber (polycarbonate enclosure).
